# A Skeptical Approach to the Management of Persistent Oral Ulceration in a Child

**DOI:** 10.1155/2018/2681723

**Published:** 2018-04-11

**Authors:** Ibrahim Kartal, Ayhan Dağdemir, Murat Elli, Levent Yıldız, Ayşegül Yılmaz

**Affiliations:** ^1^Pediatric Haematology and Oncology Department, Ondokuz Mayıs University, Samsun, Turkey; ^2^Pediatric Haematology and Oncology Department, Medipol University, Istanbul, Turkey; ^3^Pathology Department, Ondokuz Mayıs University, Samsun, Turkey; ^4^Dörtçelik Children's Hospital, Bursa, Turkey

## Abstract

The diagnosis of oral lesions is sometimes difficult due to both the clinician's limited experience with the conditions that may cause the lesions and their similar appearances, especially in children. Correctly establishing a definitive diagnosis is of major importance to clinicians who manage patients with oral mucosal diseases. In patients with Fanconi anaemia (FA), oral ulcers occur frequently, which are quite variable, and may lead to a misdiagnosis or failure to diagnose. Here, we report the case of a 15-year-old boy who was examined for squamous cell cancer of the tongue and diagnosed as having FA without any haematological manifestations. While surgery could not be done, both radiotherapy and chemotherapy had to be decreased. He died of progressive disease 6 months after the diagnosis. Unexplained ulcers in a child with a duration longer than 2 weeks should be further evaluated, especially for FA, even without the presence of anaemia.

## 1. Introduction

The diagnosis and treatment of oral lesions are often challenging due to both the clinician's limited experience with the conditions that may cause the lesions and their similar appearances. Correctly establishing a definitive diagnosis is of major importance to clinicians who manage patients with oral mucosal diseases [1].

Oral ulcers occur frequently in patients with FA and can cause anxiety due to the high risk of oral cancer. On the other hand, the FA phenotype is quite variable and may lead to a misdiagnosis or a failure to diagnose. A less severe haematological phenotype may exist in some patients as a result of somatic haematopoietic mosaicism, which can mask the diagnosis [2]. Oral ulcers or any oral lesions in a child that do not resolve within 2 weeks need to be assessed by a health care professional [1, 3] and a paediatric oncology consultation is also required. The most serious oral lesion associated with FA is oral cancer, most commonly squamous cell carcinoma (SCC) [2].

Here, we report the case of a boy who was diagnosed with SCC of the tongue while being investigated for an oral ulcer lasting for a year. He was later recognized as having FA without any haematological manifestation.

## 2. Case Report

**Figure 1:**
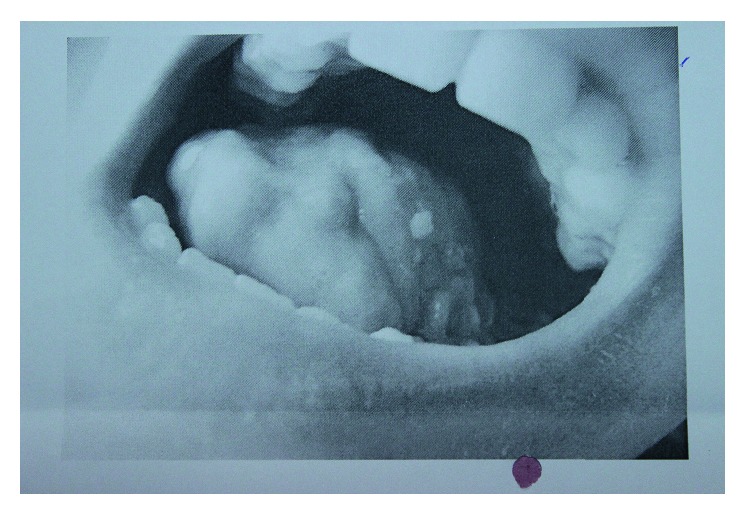
Painful oral ulcer.

**Figure 2:**
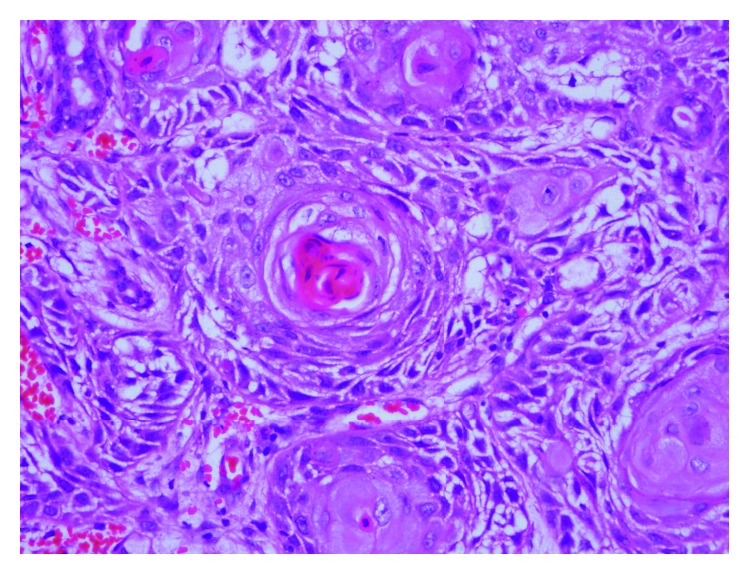
Histopathological image (haematoxylin-eosin stain; original magnification ×200). Tumour is characterized by solid islands of atypical epithelial cells with prominent nucleoli, eosinophilic cytoplasm, and large hyperchromatic nucleus in fibrotic stroma.

**Figure 3:**
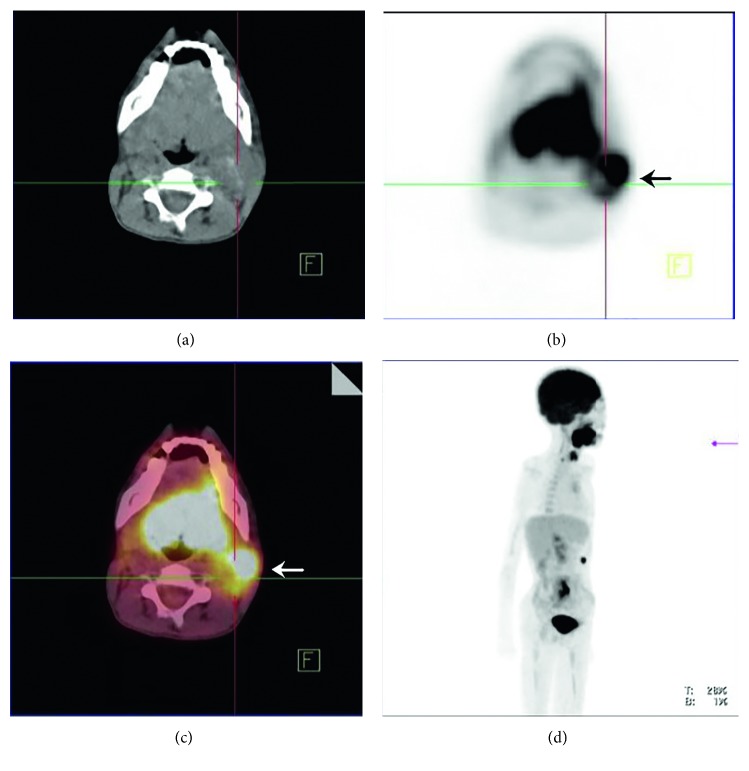
Positron emission tomography (PET)/CT with flourdeoxyglucose (FDG) showing an increased activity of SUVmax: 8, 26 in left parotid gland's posterior side, without distant metastases.

A 15-year-old boy who was receiving follow-up treatments for an operated ventricular septal defect (VSD) and ectopic kidney since he was 11 months of age developed an oral mucosal lesion that persisted for a year. The lesion did not improve during this period despite medical treatment. After this time, cryotherapy was performed because of the presence of verruca vulgaris in the lesion. However, on the follow-up examination, the lesion became larger and began to bleed during last 3 months. There was 4 × 3 cm painful necrotic lesion that reached the tonsil (Figure 1), and a pathologic lymphadenopathy was palpated on the neck. Computed tomography (CT) with contrast enhancement showed a hypodense lesion of 30 × 21 mm located posteriorly to the left parotid gland, representing an abscess or malignant lesion. Incisional biopsy from the tongue was reported as SCC (Figure 2). Positron emission tomography/CT (PET/CT) with fluordeoxyglucose (FDG) showed an increased 18F-FDG activity (SUVmax: 8, 26) in the posterior side of the left parotid gland but no distant metastasis (Figure 3). The tumour was staged as T4N2bM0. The patient had hearing loss, mental retardation, growth retardation (height and weight), and café-au-lait spots on the skin in addition to the above findings on physical examination. There was no history of smoking or alcohol consumption. Because of his phenotypical features with SCC, FA was suspected despite the absence of any significant haematological abnormality: complete blood count revealed Hb: 14.1 g/dL, MCV: 99 fL, WBC: 4230/*μ*L, and PLT: 270.000/*μ*L. FA was later confirmed by genetic analysis showing an increased chromosomal breakage with induction of mitomycin C. Cytogenetic analysis with conventional G-banding techniques revealed a normal 46 XY karyotype.

Surgery could not be done because of the locally advanced disease. The patient was treated with radiotherapy and chemotherapy, but the doses had to be decreased because of an increased susceptibility to toxicity of both treatments. He was given 2250 cGy radiotherapy to the oral cavity, and he received four cycles of reduced doses of chemotherapy consisting of cisplatin, 5-flourouracyl, and methotrexate. In spite of these treatments, he died 6 months after the diagnosis due to progressive disease.

## 3. Discussion

Recurrent oral ulcerations are commonly occurring in 1% to 10% of children [4, 5]. While the majority of cases are idiopathic, they can be associated with an underlying systemic disease including nutritional deficiencies, malignancy, haematological diseases, and inflammatory conditions. While generalized complaints may be a clue, a focused history and examination are crucial in order to reach a working differential diagnosis and to plan appropriate management [6].

In the absence of systemic features, extensive investigation for oral ulcers is rarely warranted; the child and parents can be reassured that the episodes are likely to be self-limiting. If episodes are severe, basic tests such as a complete blood count with differential and viral swabs for culture may be helpful to exclude malignancy, neutropenia, and infection [6]. For some patients, somatic haematopoietic mosaicism may have resulted in a less severe haematological phenotype masking the FA diagnosis [2]. Clinical suspicion, detailed history, and physical examination are essential, and the diagnosis can be made with molecular genetic methods.

FA is an inherited disorder associated with progressive aplastic anaemia, multiple congenital abnormalities, and a predisposition to malignancies including leukaemia and solid tumours. Pancytopenia typically manifests between the ages of 5 and 10 years, but the diagnosis can be made much earlier if there are characteristic developmental abnormalities and a family history. However, the diagnosis can be delayed in the absence of typical findings with mild haematological manifestations. The absence of birth defects or bone marrow failure does not exclude the FA diagnosis; it occasionally presents with a cancer as the first manifestation. Approximately 25% of FA patients with cancer were not recognized until they developed cancer [2]. In our case, the FA diagnosis was not warranted because of the lack of haematological parameters, which eventually lead a delayed diagnosis of SCC.

The history of this type of tumour in FA patients appears to be different compared with the healthy population. There is an increased susceptibility of the oral cavity to local predisposing factors that includes environmental toxins and viruses [2]. Sporadic head and neck SCC (HNSCC) occurs mainly in men over 60 years of age who have abused both tobacco and alcohol and who have a history of a combination of radiotherapy, chemotherapy, and surgery [7]. However, Ang et al. reported an increased incidence of females among the elderly patient population [8]. There is no underlying risk factor in FA patients, and the most common localizations of SCC are the tongue, anal and genital regions, pharynx, larynx, oral mucosa, mandible, and skin [9]. An international bibliography review on the HNSCC in FA by Lustig revealed that 13 of the 17 cases had an intraoral origin, and 9 of these cases were in the tongue. The frequency of tongue cancer in FA patients with SCC is 69%, while in non-FA patients, the incidence varies between 10% and 16% [10]. In the absence of a satisfactory screening method for oropharyngeal cancers (mainly SCC) and precancerous lesions (e.g., oral erythroleukoplakia), regular visual examinations should be carried out by a qualified general physician or dental practitioner for high-risk patients such as those with FA. Early diagnosis of these tumours may increase the chance of complete surgical excision. Regular examination of the oral cavity and oropharynx twice a year should start between the ages of 15 and 20 years of age in patients with FA, as suggested by Spanier et al. [11].

## 4. Conclusion

Physicians should be cautious in the diagnosis and treatment of oral ulcers. It is important to understand that oral manifestations may represent only a part of a large problem. Squamous cell carcinoma of the oral cavity can mimic a variety of benign conditions occurring at multiple sites. Therefore, a careful soft tissue examination and medical history should be performed at each dental or medical appointment. Any unexplained ulcer that is present longer than 2 weeks should be further evaluated and biopsied. Dermatologists, dentists, and paediatric haematologists and oncologists should be in close contact in the management of these patients.
